# Comparison of short- and long-term outcomes among laparoscopic, robotic-assisted, and transanal total mesorectal excision procedures in patients with rectal cancer: a propensity score-matching analysis

**DOI:** 10.1007/s10151-025-03204-5

**Published:** 2025-08-18

**Authors:** G.-Y. Chen, C.-K. Liao, J.-F. You, C.-C. Lai, S.-H. Huang

**Affiliations:** 1 Department of Surgery, Chang Gung Memorial Hospital, Linkou, No. 5, Fuxing St., Guishan Dist., Taoyuan, 333 Taiwan; 2 Division of Colon and Rectal Surgery, Department of Surgery, Chang Gung Memorial Hospital, Linkou, No. 5, Fuxing St., Guishan Dist., Taoyuan, 333 Taiwan; 3 School of Medicine, Chang Gung University, No. 259, Wenhua 1St Road, Guishan Dist., Taoyuan, 333 Taiwan

**Keywords:** Total mesorectal excision, Rectal cancer, Surgery, Robotics, Laparoscopy, Transanal total mesorectal excision

## Abstract

**Background:**

Total mesorectal excision (TME) remains the oncologic standard for rectal cancer surgery; however, technical challenges persist in the minimally invasive treatment of low rectal cancer. Transanal TME (TaTME) and robotic TME were developed to overcome the limitations of laparoscopic TME in confined pelvic spaces. Despite promising results, comparative evidence among these approaches remains limited and heterogeneous. To address this gap, we conducted a propensity score-matched analysis to evaluate and compare the clinical and oncologic outcomes of TaTME, robotic TME, and laparoscopic TME in patients with rectal cancer treated at a high-volume tertiary center.

**Methods:**

This retrospective study included patients with rectal cancer who underwent restorative proctectomy between 2015 and 2021. Propensity score matching was used to balance demographic, clinical, and treatment variables across the three groups. Outcomes were analyzed using standard statistical methods.

**Results:**

After matching, 240 patients were included (40 TaTME, 40 robotic TME, and 160 laparoscopic TME). TaTME and robotic TME demonstrated significantly lower overall complication rates than laparoscopic TME (27.5% versus 20.0% versus 39.4%, *p* = 0.033). The circumferential resection margin positivity rate was highest in the laparoscopic group (10.6% versus 0% versus 2.5%, *p* = 0.031). However, 5-year overall survival (82.5% versus 85.0% versus 88.1%, *p* = 0.251), disease-free survival (75.0% versus 72.5% versus 73.8%, *p* = 0.772), local recurrence (17.5% versus 12.5% versus 24.7%, *p* = 0.488), and distal metastasis (17.5% versus 22.5% versus 25.2%, *p* = 0.694) did not significantly differ among groups.

**Conclusions:**

All three minimally invasive TME techniques achieved comparable long-term oncologic outcomes. Surgical approach should be tailored on the basis of surgeon expertise and patient-specific factors.

## Introduction

The management of locally advanced rectal cancer (LARC) has evolved to include neoadjuvant therapy, surgery, and adjuvant treatment, reducing recurrence risk [1]. Among these therapies, surgical precision remains key to curative outcomes.

Laparoscopic TME (LapTME), the pioneering minimally invasive technique, has demonstrated noninferior oncologic outcomes compared with open TME [2–4]. It offers improved short-term outcomes, such as shorter hospital stays, fewer wound-related complications, and faster functional recovery [4, 5]. However, performing LapTME for mid- and low rectal tumors presents significant technical challenges, particularly in patients with a narrow pelvic cavity, bulky tumors, male gender, or obesity [4, 6]. To overcome these limitations, transanal TME (TaTME) and robotic-assisted TME (robotic TME) were introduced.

TaTME was developed to overcome the challenges associated with pelvic dissections for low rectal cancer, which are often difficult to manage using traditional transabdominal approaches. The improved visualization and access afforded by TaTME enable more precise dissections, potentially resulting in superior specimen quality and better circumferential and distal resection margins, as supported by several large trials and meta-analyses [7–10]. However, the clinical outcomes of TaTME have shown mixed results in the literature. Norwegian study reported inferior oncological outcomes for TaTME, which may be attributed to the early enrollment period when surgical teams had not yet overcome the learning curve [11]. Subsequently, studies have demonstrated that when performed by experienced teams, TaTME achieves oncological outcomes comparable to conventional LapTME, without showing particularly significant advantages [12].

In some studies, robotic TME offers enhanced three-dimensional (3D) visualization, stable imaging, and flexible instrumentation [13–15]. However, the outcomes assessed in these studies vary, and their methodological quality is inconsistent [16]. The REAL trial demonstrated that robotic surgery offers superior short-term outcomes and improved locoregional control and disease-free survival compared with LapTME [17–19]. In contrast, the ROLLAR trial revealed no significant advantages of robotic TME over LapTME across multiple short-term outcomes, including intraoperative and postoperative complications, surgical plane quality, 30-day mortality, bladder and sexual dysfunction, and conversion rates [20]. Furthermore, long-term outcomes appeared comparable between the two approaches [21, 22].

Few randomized controlled trials (RCTs) have compared all three minimally invasive approaches in one study. Most focus on short-term outcomes or specific tumor locations [23–25]. For instance, the RESET trial excluded patients requiring abdominoperineal resection (APR), and the patients in the lapTME group had favorable tumor locations, introducing potential selection bias against low rectal cancer [25]. Given these considerations, we conducted a retrospective, single-center study to compare the clinical and short- and long-term outcomes of three minimally invasive techniques, using propensity score matching to ensure balanced rectal tumor locations at a high-volume referral center.

## Materials and methods

### Patient selection

This retrospective study reviewed patients who underwent restorative proctectomy for rectal cancer at Chang Gung Memorial Hospital between January 2015 and December 2021. The study was approved by the Institutional Review Board (approval no. 202200885B0), and the requirement for informed consent was waived owing to the use of deidentified data.

**Fig. 1:**
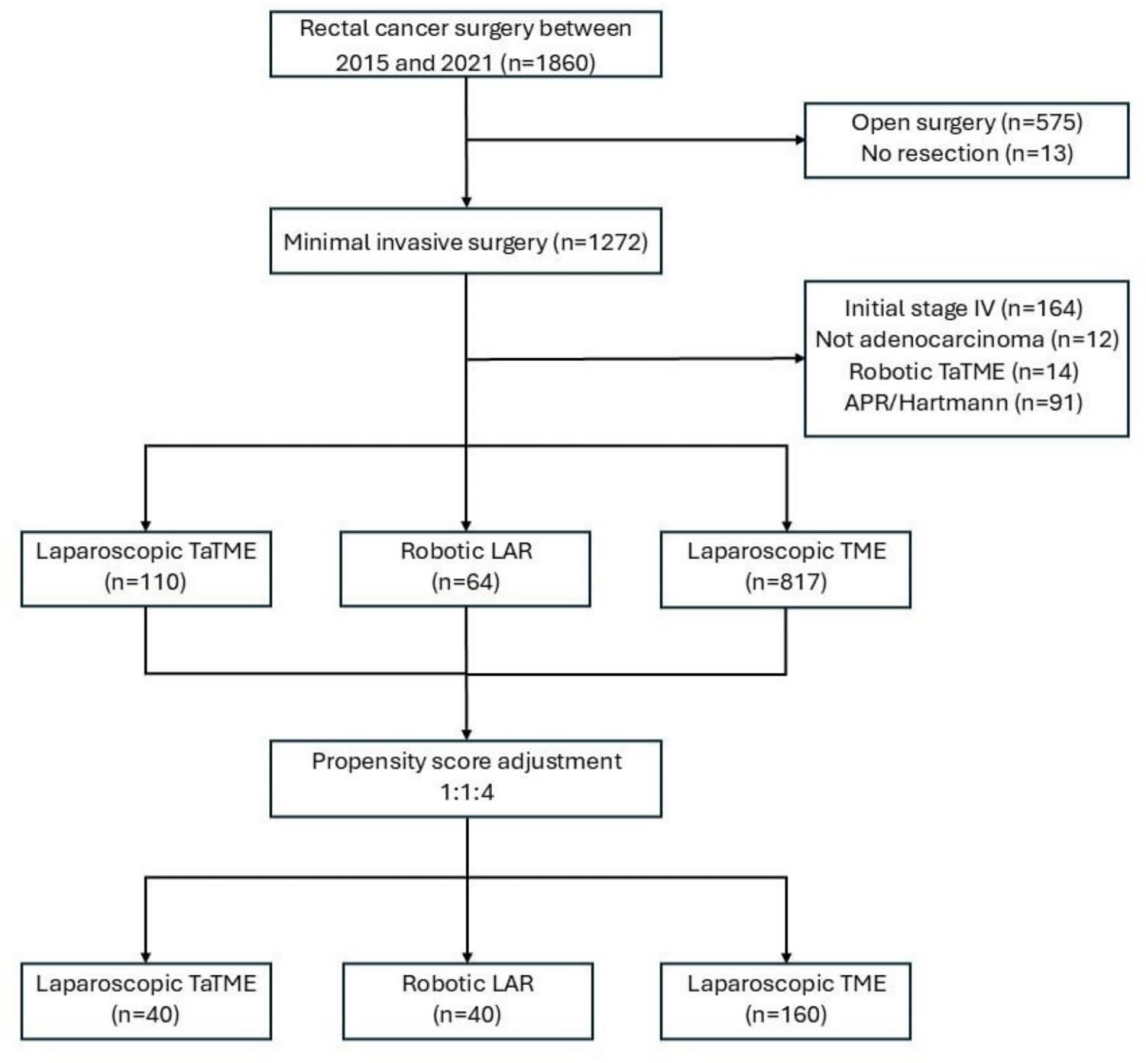
Flowchart illustrating the clinical patient selection in this study

Eligible participants were identified among rectal cancer patients who had completed a comprehensive diagnostic workup, including colonoscopy with biopsy, chest–pelvic computed tomography (CT), rectal magnetic resonance imaging (MRI), and endorectal ultrasound if needed. To define the study population, we established specific inclusion and exclusion criteria. We included patients with biopsy-confirmed rectal adenocarcinoma located within 15 cm of the anal verge, deemed suitable for total mesorectal excision (TME) and anastomosis. Tumor location was assessed using rigid proctoscopy to measure the distance from the anal verge. We excluded patients who required conventional open surgery, those in whom tumor resection was not feasible, patients with stage IV disease at initial diagnosis, and those who underwent alternative surgical approaches, including robotic transanal TME (TaTME), abdominoperineal resection (APR), or Hartmann’s procedure. A detailed flowchart of the patient selection process is provided in Fig. 1.

#### Assessment and treatment protocol

The preoperative evaluation included a comprehensive physical examination, digital rectal examination, colonoscopy with biopsy, and CT imaging of the chest, abdomen, and pelvis. Pelvic MRI delineated locoregional tumor characteristics. Serum carcinoembryonic antigen (CEA) levels were measured during the routine assessment.

Tumor staging was conducted according to the 8th edition of the Union for International Cancer Control (UICC) tumor–node–metastasis (TNM) classification system [26]. A multidisciplinary team (MDT) reviewed and finalized treatment strategies. On the basis of tumor stage and MDT recommendations, the neoadjuvant treatment approach was determined.

The selection between TaTME and robotic TME was guided by surgeon preference, tumor-specific characteristics, and individual patient considerations. The decision to create a protective stoma was also made at the discretion of the operating surgeon.

#### Data collection

Patient data were extracted from our institution’s electronic medical records. Key preoperative metrics included demographic data, clinical parameters, laboratory results (serum albumin and CEA levels), and tumor characteristics (location and TNM stage).

The analysis covered clinical and procedural factors such as neoadjuvant and adjuvant therapies, surgical details (blood loss, stoma creation, conversion to open surgery), and anastomotic techniques. Pathological evaluation included tumor histology, differentiation, lymphovascular and perineural invasion, circumferential and distal resection margins (CRM and DRM), and lymph node yield. Margins were considered positive if tumor cells were present within 1 mm of the specimen edge.

Treatment outcomes were assessed using the Clavien–Dindo (C–D) classification for surgical complications and by recording mortality. Severe complications were defined as Clavien–Dindo grade III or higher [27]. Long-term oncologic outcomes included overall survival (OS), disease-free survival (DFS), local recurrence (LR), and distant metastasis (DM).

#### Follow-up

In line with our department’s standardized protocol, a structured surveillance program was implemented for all participants. Clinical evaluations were conducted every 3–6 months during the first 3 years post-surgery, then annually thereafter. Each visit included a physical examination, CEA testing, and imaging to monitor for recurrence.

The 5-year follow-up protocol incorporated annual whole-body CT scans (chest to pelvis) and colonoscopic examinations. If clinical signs suggested recurrence, diagnostic evaluations were performed ahead of schedule.

OS was defined from the date of tumor resection to death or final chart review. DFS was measured from surgery to the first recurrence, death, or last follow-up. Data collection continued through 30 December 2024, to support comprehensive survival analysis.

### Statistical analysis

Summary statistics are reported as median with interquartile range (IQR). Quantitative variables were compared using one-way analysis of variance (ANOVA), while categorical variables are presented as frequency and percentage, analyzed via chi-squared or Fisher’s exact test as appropriate. Survival outcomes were assessed using Kaplan–Meier curves and compared with the log-rank test. Univariate logistic regression was used to identify risk factors for postoperative complications.

Propensity scores were calculated using logistic regression on the basis of key preoperative variables, including gender, tumor location, T stage, and the use of neoadjuvant chemotherapy, radiotherapy, and adjuvant chemotherapy. Variables with significant baseline differences among surgical groups informed the matching criteria. An initial 1:1 PSM was performed between TaTME and robotic surgery patients. The matched cohort was then combined and subjected to 1:2 PSM with the LapTME group to achieve balanced baseline characteristics across all groups. Statistical significance was set at *p* < 0.05. All analyses were performed using SPSS version 26 (IBM Corp., New York, NY, USA).

## Results

### Patient characteristics before and after PSM adjustment

Table 1 compares baseline and preoperative clinicopathological characteristics before and after propensity score matching (PSM). A total of 987 patients were included prior to matching: 110 in the TaTME group, 64 in the robotic TME group, and 817 in the LapTME group. In the unmatched cohort, significant differences were observed. Tumor location varied notably, with TaTME patients having a higher proportion of low rectal tumors compared with the other groups (*p* < 0.001). Pathological T-stage distribution also differed significantly (*p* = 0.001). The TaTME group received neoadjuvant therapy most frequently, followed by the robotic TME and LapTME groups.

**Table 1:** Basic characteristics of patients with rectal cancer who underwent restorative proctectomy before and after propensity score matching

	Before propensity score matching				After propensity score matching			
	TaTME (*n* = 110)	Robotic LAR (*n* = 64)	LapTME (*n* = 817)	*p*-value	TaTME (*n* = 40)	Robotic LAR (*n* = 40)	LapTME (*n* = 160)	*p*-value
Age (years)	59.5 (17)	60.5 (19)	63 (16)	0.098	59.5 (17)	60.5 (14)	61 (14)	0.333
Gender								
Male	81 (73.6)	41 (64.1)	494 (60.5)	0.027	26 (65)	25 (72.5)	101 (63.1)	0.969
Female	29 (26.4)	23 (35.9)	323 (39.5)		14 (35)	15 (27.5)	59 (36.9)	
BMI (kg/m^2^)	24.5 (4.25)	23 (4)	24(4)	0.244	24.6 (5.5)	24.1 (4)	24 (4)	0.393
Distance from AV (cm)	5 (2)	9.5 (5)	10 (4)	< 0.001	7 (3)	6 (6)	8 (5)	0.02
Tumor location								
Upper rectum	2 (1.8)	12 (18.8)	250 (30.6)	< 0.001	2 (5)	3 (7.5)	16 (10)	0.215
Middle rectum	30 (27.3)	32 (50)	492 (60.2)		27 (67.5)	18 (45)	98 (61.3)	
Lower rectum	78 (70.9)	20 (31.3)	75 (9.2)		11 (27.5)	19 (47.5)	46 (28.7)	
Albumin (g/dL)								
< 3.5	5 (4.6)	3 (4.7)	35 (4.3)	0.510	4 (10)	2 (5)	4 (2.5)	0.101
≥ 3.5	103 (95.4)	61 (95.3)	782 (95.7)		36 (90)	38 (95)	156 (97.5)	
CEA (ng/ml)								
< 5	91 (82.7)	51 (79.7)	630 (77.1)	0.386	33 (82.5)	36 (90)	133 (83.1)	0.539
≥ 5	19 (17.3)	13 (20.3)	187 (22.9)		7 (17.5)	4 (10)	27 (16.9)	
ASA score								
2	47 (42.7)	25 (39.1)	311 (38.1)	0.843	14 (35)	17 (42.5)	57 (35.6)	0.702
3	63 (57.3)	39 (60.9)	506 (61.9)		26 (75)	23 (57.5)	103 (64.4)	
pT-stage								
T0	11 (10)	3 (4.7)	24 (2.9)	0.001	3 (7.5)	2 (5)	11 (6.9)	0.221
T1	11 (10)	6 (9.4)	143 (17.5)		6 (15)	6 (15)	26 (16.3)	
T2	29 (26.4)	16 (25)	165 (20.2)		7 (17.5)	10 (25)	34 (21.3)	
T3	53 (48.2)	38 (59.4)	418 (51.2)		21 (52.5)	21 (52.5)	60 (37.5)	
T4	6 (5.5)	1 (1.6)	67 (8.2)		3 (7.5)	1 (2.5)	29 (18.1)	
pN-stage								
N0	66 (60)	41 (64.1)	496 (60.7)	0.726	25 (62.5)	26 (65)	81 (50.6)	0.433
N1	33 (30)	15 (23.4)	209 (256)		9 (22.5)	9 (22.5)	49 (30.6)	
N2	11 (10)	8 (12.5)	112 (13.7)		6 (15)	5 (12.5)	30 (18.8)	
Tumor size (cm)	3.0 (2.2)	3.6 (2.7)	3.5 (2.4)	0.008	3.0 (2.2)	3.5 (2.6)	3.4 (2.4)	0.089
Neoadjuvant treatment								
Yes	88 (20)	38 (59.4)	393 (48.1)	< 0.001	27 (67.5)	24 (60)	109 (68.1)	0.617
No	22 (80)	26 (40.6)	424 (51.9)		13 (32.5)	16 (40)	51 (31.9)	
Neoadjuvant RT	52 (47.3)	15 (23.4)	77 (9.4)	< 0.001	16 (37.5)	10 (25)	39 (24.4)	0.236
Neoadjuvant chemotherapy	28 (25.5)	11 (17.2)	61 (7.5)	< 0.001	6 (15)	8 (20)	29 (18.1)	0.838
Adjuvant chemotherapy	68 (61.8)	26 (40.6)	338 (41.4)	< 0.001	22 (55)	18 (45)	88 (55)	0.512
Chemotherapy with Oxaliplatin	47 (42.7)	19 (29.7)	158 (19.3)	< 0.001	19 (47.5)	14 (35)	73 (45.6)	0.431

*LapTME* laparoscopic total mesorectal excision, *TaTME* transanal total mesorectal excision, *BMI* body mass index, *AV* anal verge, *ASA* American Society of Anesthesiologists

After PSM, 40 patients were included in both the TaTME and robotic TME groups and 160 in the LapTME group. Matching achieved balance across most baseline variables, except for tumor distance from the anal verge, which remained significantly different (*p* = 0.02). No significant differences were observed in gender, tumor location, pT stage, or neoadjuvant chemotherapy, radiotherapy, and adjuvant chemotherapy use.

#### Operative parameters and short-term outcomes

Operative parameters are summarized in Table 2. Operative time was significantly longer in the TaTME and robotic TME groups compared with LapTME (*p* < 0.001), while blood loss was similar across groups (*p* = 0.639). The rate of diverting stoma creation differed significantly (*p* < 0.001), being highest in the TaTME group (80%), followed by robotic TME (47.5%) and LapTME (37.5%). No conversions occurred in the TaTME group. The robotic TME group had one conversion due to severe adhesions. In the LapTME group, one conversion was due to adhesions and four to advanced disease. The stapled anastomosis was more frequently used in LapTME (*p* < 0.001). Natural orifice specimen extraction was more common in TaTME and robotic TME than in LapTME (75% versus 42.5% versus 12.5%, *p* < 0.001).

**Table 2:** Post-matching of operative parameters among patients with rectal cancer who underwent restorative proctectomy

	TaTME (*n* = 40)	Robotic LAR (*n* = 40)	LapTME (*n* = 160)	*p* value	*p* TaTME versus robotic LAR	*p* TaTME versus LapTME	*p* Robotic LAR versus LapTME
Operative time (minutes)	335.5 (119)	347 (181)	260.5 (106)	< 0.001	0.214	0.001	< 0.001
Blood loss	50 (50)	30 (30)	50 (30)	0.639			
< 100 ml	29 (72.5)	32 (80)	132 (82.5)	0.361			
≥ 100 ml	11 (27.5)	8 (20)	28 (17.5)				
Diverting stoma							
Yes	32 (80)	19 (47.5)	60 (37.5)	< 0.001	0.012	< 0.001	0.01
No	8 (20)	21 (52.5)	100 (62.5)				
Conversion							
Yes	0	1 (2.5)	5 (3.1)	0.527			
No	40 (100)	39 (97.5)	155 (96.9)				
Anastomosis methods							
No	2 (5)	4 (10)	0	< 0.001	0.569	< 0.001	< 0.001
Hand sewn	12 (30)	9 (22.5)	3 (1.9)				
Staples	26 (65)	27 (67.5)	157 (98.1)				
Specimen extraction methods							
NOSE	30 (75)	17 (42.5)	20 (12.5)	< 0.001	0.003	< 0.001	< 0.001
Pfannenstiel incision	0	0	4 (2.5)				
Other abdominal incision	10 (25)	23 (57.5)	136 (85)				

*LapTME* laparoscopic total mesorectal excision, *TaTME* transanal total mesorectal excision, *NOSE* natural orifice specimen retraction

Postoperative outcomes are presented in Table 3. Median hospital stay and recovery milestones were comparable among groups. However, overall complication rates differed significantly (*p* = 0.033), being highest in LapTME (39.4%), followed by TaTME (27.5%) and robotic TME (20%). Complication grade distribution also varied (*p* = 0.026), with major complications more frequent in the LapTME group (*p* = 0.045). Specifically, nine patients (5.6%) developed anastomotic leakage, four (2.5%) experienced ileus, and two (1.25%) had intraabdominal infections. In the robotic TME group, two patients (5%) had major complications—one with anastomotic leakage and one with a ureteral injury. Major complication rates were significantly higher in LapTME than TaTME (*p* = 0.031), but comparable to robotic TME group (*p* = 0.375). Minor complication rates showed no significant difference (*p* = 0.161). Specific complications such as ileus (*p* = 0.383), anastomotic leak (*p* = 0.556), intraabdominal infection (*p* = 0.793), and bladder dysfunction (*p* = 0.556) were similar among groups. Reoperation rates were low, with no significant differences. Permanent stoma formation rates were also comparable (17.5% versus 17.5% versus 12%, *p* = 0.514).

**Table 3:** Post-matching of short-term outcomes among patients with rectal cancer who underwent restorative proctectomy

	TaTME (*n* = 40)	Robotic LAR (*n* = 40)	LapTME (*n* = 160)	*p* value	*p* TaTME versus robotic LAR	*p* TaTME versus LapTME	*p* Robotic LAR versus LapTME
Hospital stay (days)	8 (4)	7 (4)	7 (5)	0.257			
First flatus passage	2 (1)	2 (2)	2 (2)	0.309			
First stool passage (days)	3 (2)	3 (2)	4 (4)	0.013	0.880	0.663	0.439
Tolerated liquid diet (days)	3 (3)	2 (3)	3 (4)	0.071			
Tolerated soft diet (days)	5 (3)	4 (3)	5 (4)	0.313			
Remove Foley day (days)	4 (3)	4 (4)	5 (3)	0.224			
Overall complication							
Yes	11 (27.5)	8 (20)	63 (39.4)	0.033	0.431	0.042	0.022
No	29 (72.5)	32 (80)	97 (60.6)				
Clavien–Dindo classification							
I	0	0	10 (6.3)	0.026	0.183	0.034	0.046
II	11 (27.5)	6 (15)	38 (23.8)				
III	0	2 (5)	10 (6.3)				
IV	0	0	4 (2.5)				
V	0	0	1 (0.6)				
Major complication (C–D grade ≥ 3)	0	2 (5)	15 (9.4)	0.045	0.152	0.031	0.375
Minor complication (C–D grade < 3)	11 (27.5)	6 (15)	48 (30.1)	0.161			
Complication type							
Ileus	4 (10)	1 (2.5)	10 (6.3)	0.383			
Anastomosis leak	1 (2.5)	1 (2.5)	9 (5.6)	0.556			
IAI	6 (15)	4 (10)	21 (13.1)	0.793			
Bladder dysfunction	1 (2.5)	3 (7.5)	11 (6.9)	0.556			
Others	1 (2.5)	1 (2.5)	15 (9.4)	0.147			
Reoperation							
Leakage	0	1 (2.5)	5 (3.1)	0.718			
Bowel obstruction	0	0	2 (1.3)				
Others	0	1 (2,5)	5 (3.1)				
Permanent stoma	7 (17.5)	7 (17.5)	19 (12)	0.514			

*LapTME* laparoscopic total mesorectal excision, *TaTME* transanal total mesorectal excision, *IAI* intraabdominal infection

Table 4 summarizes risk factors for postoperative complications. No significant predictors were found for major complications. However, robotic TME (OR = 0.403, *p* = 0.041) and TaTME (OR = 0.258, *p* = 0.010) were protective against overall complications compared with LapTME. Male sex (OR = 1.049, *p* = 0.043) and mid rectal tumor location (OR = 3.622, *p* = 0.026) were associated with increased risk. Among neoadjuvant treatments, radiotherapy significantly increased the risk (OR = 2.580, *p* = 0.011), while chemotherapy did not. Other factors—including tumor location, ASA score, pT stage, and pN stage—showed no significant association with overall complications.

**Table 4:** Summary of the risk factors for postoperative complications

Major complications (C–D grade ≥ 3)			Overall complication		
	OR	*p*-value		OR	*p*-value
LapTME	1		LapTME	1	
Robotic LAR	0.998	0.582	Robotic LAR	0.403	0.041
TaTME	0.529	0.431	TaTME	0.298	0.010
Age > 75 years	1.431	0.529	Age > 75 years	1.639	0.255
Male sex	1.340	0.617	Male sex	1.043	0.043
BMI > 27	1.329	0.629	BMI > 27	1.682	0.122
Upper rectum	1		Upper rectum	1	
Middle rectum	1.129	0.900	Middle rectum	3.622	0.026
Lower rectum	1.489	0.635	Lower rectum	1.571	0.393
ASA score			ASA score		
2	1		2	1	
3	0.984	0.989	3	0.885	0.688
pT-stage			pT-stage		
0 + 1 + 2	1		0 + 1 + 2	1	
3	1.008	0.991	3	0.865	0.676
4	2.012	0.367	4	1.129	0.801
pN-stage			pN-stage		
N0	1		0	1	
N1	0.441	0.244	1	0.973	0.943
N2	0.341	0.238	2	1.233	0.643
Diverting stoma	0.672	0.421	Diverting stoma	0.75	0.056
Neoadjuvant chemotherapy	1.945	0.722	Neoadjuvant chemotherapy	0.303	0.067
Neoadjuvant radiotherapy	0.225	0.423	Neoadjuvant radiotherapy	2.560	0.01

*LapTME* laparoscopic total mesorectal excision, *TaTME* transanal total mesorectal excision, *OR* odds ratio

### Histopathological parameters

The histopathological outcomes are summarized in Table 5. A pathological complete response was observed in five patients (12.5%) in the TaTME group, six patients (15%) in the robotic TME group, and 16 patients (10%) in the LapTME group (*p* = 0.799). However, the rate of circumferential resection margin (CRM) positivity was significantly higher in the LapTME group compared with the other two groups (10.6% versus 0% versus 2.5%, *p* = 0.031). The three groups exhibited comparable histological types (*p* = 0.967) and tumor grades (*p* = 0.636). Similarly, no significant differences were observed among the groups in terms of lymphovascular invasion (*p* = 0.133), perineural invasion (*p* = 0.527), distal resection margin (DRM) positivity (*p* = 0.322), or lymph node yield (*p* = 0.268).

**Table 5:** Post-matching pathological finding among patients with rectal cancer who underwent restorative proctectomy

	TaTME (*n* = 40)	Robotic LAR (*n* = 40)	LapTME (*n* = 160)	*p* value	*p* TaTME versus robotic LAR	*p* TaTME versus LapTME	*p* Robotic LAR versus LapTME
pCR	5 (12.5)	6 (15)	16 (10)	0.799			
Histology type							
Adenocarcinoma	39 (97.5)	38 (95)	151 (94.4)	0.967			
Signet ring cell/Mucinous	1 (2.5)	2 (5)	9 (5.6)				
Histology grade							
Grade I/II	35 (87.5)	35 (87.5)	146 (91.3)	0.636			
Grade III	4 (10)	3 (7.5)	12 (7.5)				
Unclassified	1 (2.5)	2 (5)	2 (1.3)				
Lymphovascular invasion							
Positive	5 (12.5)	8 (20)	50 (31.3)	0.133			
Negative	34 (85)	31 (77.5)	108 (67.5)				
Unknown	1 (2.5)	1 (2.5)	2 (1.3)				
Perineural invasion							
Positive	5 (12.5)	10 (25)	39 (24.4)	0.527			
Negative	34 (85)	29 (72.5)	119 (74.4)				
Unknown	1 (2.5)	1 (2.5)	2 (1.3)				
CRM							
Positive	1 (2.5)	0	17 (10.6)	0.031	n/a	0.034	0.027
Negative	39 (97.5)	40 (100)	143 (89.4)				
Distal resection margin, length	1.7 (1.7)	1.8 (1.2)	1.9 (1.7)	0.368			
Distal resection margin							
Positive	1 (2.5)	0	7 (4.4)	0.322			
Negative	39 (97.5)	40 (100)	153 (95.6)				
Lymph node yield	25 (16)	22 (17)	24 (15)	0.268			
R1 resection	4 (9.5)	0	13 (8.1)	0.148			

### Long-term outcomes

**Fig. 2:**
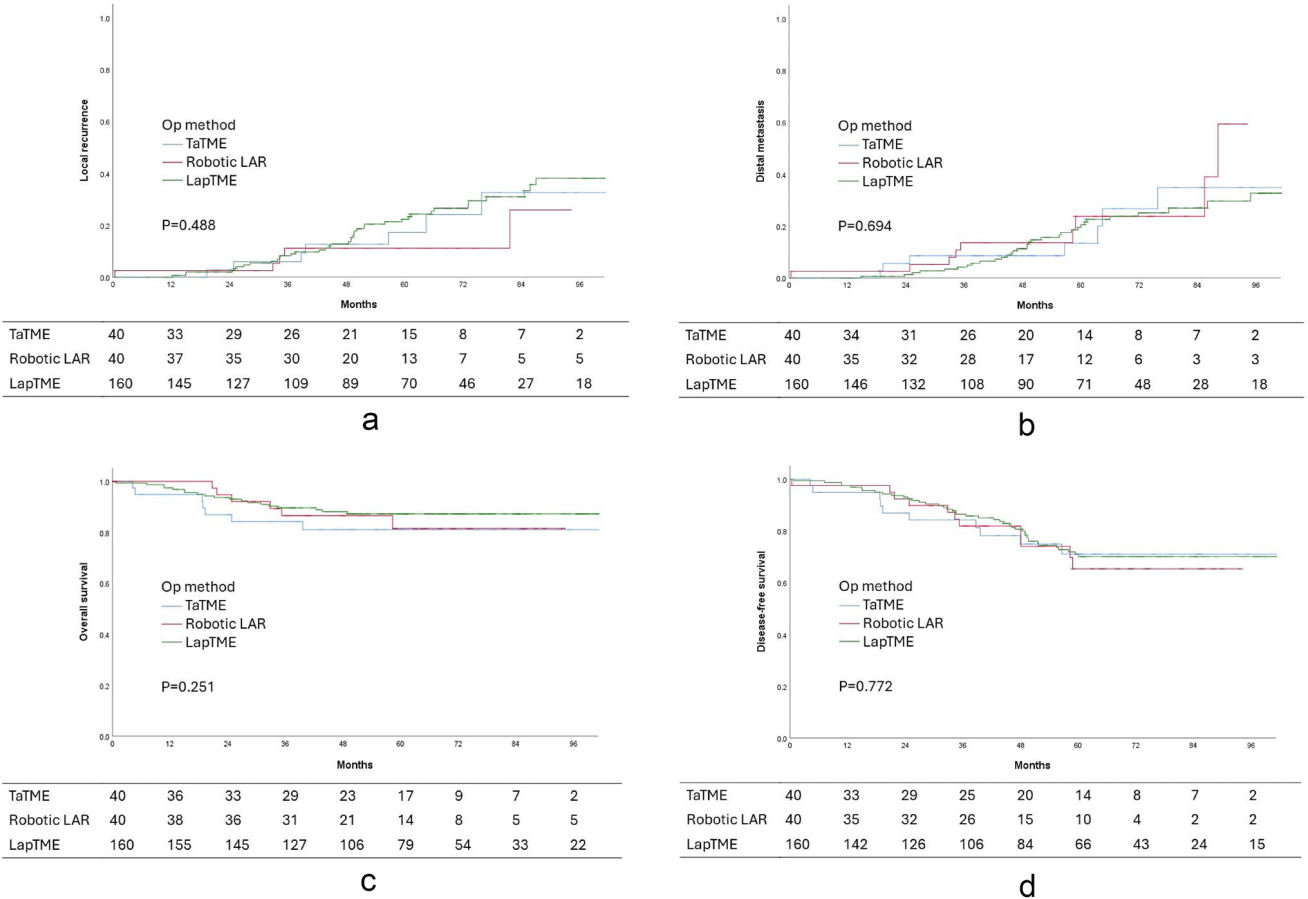
**a** Kaplan–Meier survival curves for local recurrence. **b** Kaplan–Meier survival curves for distal metastasis. **c** Kaplan–Meier survival curves for overall survival. **d** Kaplan–Meier survival curves for disease-free survival

The median follow-up durations were 55.1, 48.0, and 59.7 months for the TaTME, robotic TME, and LapTME groups, respectively (*p* = 0.07). Five-year LR rates were 17.5%, 12.5%, and 24.7% (*p* = 0.488), and 5-year DM rates were 17.5%, 22.5%, and 25.2% (*p* = 0.694) for TaTME, robotic TME, and LapTME, respectively. Five-year overall survival (OS) rates (82.5% versus 85.0% versus 88.1%, *p* = 0.251) and 5-year disease-free survival (DFS) rates (75.0% versus 72.5% versus 73.8%, *p* = 0.772) did not reach statistical significance as well (Fig. 2).

## Discussion

This study compared the clinical and oncological outcomes of TaTME, robotic TME, and LapTME in post-PSM patients with similar tumor distribution at a high-volume referral center. The PSM analysis demonstrated equivalent oncological outcomes among the three minimally invasive techniques. Although LapTME was associated with higher rates of postoperative overall complications and circumferential resection margin (CRM) positivity compared with TaTME and robotic TME, the short- and long-term outcomes were comparable across all three minimally invasive approaches.

Regarding operative parameters, the median operative time for LapTME was significantly shorter than that of TaTME and robotic TME. A previous study reported reduced operative time for TaTME compared with conventional LapTME [28]. The operative time can be reduced through simultaneous transabdominal and transanal dissection by two surgical teams [29]. The discrepancy between our findings and earlier studies may be due to the higher proportion of hand-sewn anastomoses and diverting stomas in the TaTME group. Robotic surgery, while offering enhanced precision and control, typically requires longer operative time owing to procedural steps such as docking, instrument calibration, and port placement optimization [20, 30]. The absence of haptic feedback also necessitates more deliberate and systematic instrument handling. Moreover, the high cost of robotic systems may impose additional financial burdens on patients [22]. Therefore, the suitability of the robotic approach should be carefully evaluated preoperatively.

Conversion remains a significant challenge in laparoscopic surgery. A key advantage of robotic and transanal approaches is their lower conversion rates, attributed to enhanced access within the pelvic cavity [28, 31]. The ROLARR trial reported conversion rates of 8.1% for robotic TME and 12.2% for laparoscopic TME, though the difference was not statistically significant [20]. These higher rates may reflect early experiences during the learning curve. In our study, conversion rates were low and comparable across all three techniques. This aligns with findings from a previous RCT involving only experienced surgeons, which reported conversion rates of 1.9% for TaTME, 4.6% for robotic TME, and 3.7% for LapTME [23]. These results highlight the importance of performing complex procedures beyond the learning curve to ensure optimal surgical outcomes.

In our study, the rate of postoperative complications was significantly higher in the LapTME group than in the TaTME and robotic TME groups. The odds ratios indicated a reduced overall risk of complications for TaTME (OR = 0.298) and robotic TME (OR = 0.403). Similar comparisons of short-term outcomes have been reported in several studies [7, 12, 13, 29]. The higher rate of major complications observed in both the robotic TME and LapTME groups in our study may be secondary to the lower rate of protective stoma formation (TaTME: 80%, robotic TME: 47.5%, LapTME: 37.5%). Consequently, multivariate regression analysis showed no significant difference after accounting for diverting stomas. The higher rate of major complications observed in both the robotic TME and LapTME groups in our study may be secondary to the lower rate of protective stoma formation. While multivariate regression analysis showed no significant difference after accounting for diverting stomas, it is important to note that the elevated incidence of major complications in these groups may not be solely attributable to the lower rate of diverting stomas. The absence of haptic feedback in robotic systems can impede tactile perception during dissection, while prolonged operative time may also elevate the risk of tissue injury. Additionally, the higher rate of stoma formation specifically in lower rectal tumors may comparatively lead to a higher complication rate in mid rectal tumors. This observation is further supported by our multivariate analysis, which identified middle rectal tumor location as an independent risk factor for complications (OR = 3.622, *p* = 0.026), underscoring its significant clinical relevance.

Anastomotic leakage remains a critical complication in TME procedures. The limited space in the deep pelvis during laparoscopic surgery often requires multiple applications of linear staplers, which increases the risk of leakage [32]. In contrast, TaTME and robotic TME improve pelvic visualization and anatomical precision, potentially reducing the need for staplers. While the leakage rate in the LapTME group was slightly higher than in the other two groups, it did not reach statistical significance, likely owing to smaller sample sizes in the TaTME and robotic TME cohorts. This difference may also be influenced by the higher frequency of diverting stoma creation in TaTME and robotic TME patients [33, 34]. Notably, our analysis showed that preoperative neoadjuvant radiotherapy significantly increased the risk of postoperative complications (OR = 2.560), consistent with previous findings [35, 36]. Although radiotherapy improves oncologic outcomes, it can damage surrounding tissues, elevating the risk of anastomotic complications and the need for permanent stomas [37, 38]. The observed higher complication rate in mid rectal tumors may be due to the routine use of protective stomas in low rectal cases. Despite these differences, hospital stay and functional recovery times were comparable across all three minimally invasive approaches, supporting their overall reliability.

The quality of rectal surgery is often assessed through pathological parameters, particularly the circumferential resection margin (CRM) positivity rate, a key predictor of pelvic recurrence and distant metastasis [12, 23, 28]. In our study, CRM positivity rates were 2.5% in TaTME, 0% in robotic TME, and 10.6% in LapTME, with the LapTME group showing significantly higher rates than the other two. A meta-analysis by Martínez-Pérez et al. reported that LapTME was associated with higher rates of incomplete mesorectal excision than open surgery (risk ratio 1.31, 95% CI 1.05–1.64) [39]. This finding may be due to the technical limitations of conventional laparoscopy, including reduced instrument maneuverability, which can compromise precise dissection within the narrow pelvis. The transanal approach was developed to address these challenges, offering improved visualization and access. A meta-analysis by Aubert et al. demonstrated that TaTME significantly reduced CRM involvement compared with LapTME (OR 0.48, 95% CI 0.27–0.86, *p* = 0.01) [7]. Similarly, the International TaTME Registry reported a low CRM positivity rate of 2.4%, aligning with our findings [40]. Robotic TME offers enhanced dexterity and facilitating accurate dissection in confined spaces. The REAL trial found robotic TME superior to LapTME in achieving negative CRM margins (4.0% versus 7.2%; difference −3.2%, 95% CI −6.0% to −0.4%; *p* = 0.023), especially in complex cases involving male patients, low tumors, and T3–T4a stages [17]. No significant differences were observed among the three groups regarding distal resection margin (DRM) and R1 resection rates, suggesting that all techniques achieved high oncological quality in these parameters.

Long-term oncological outcomes are a critical benchmark for evaluating surgical interventions in cancer patients. A Norwegian study previously reported a 9.5% local recurrence (LR) rate among patients undergoing TaTME at a median follow-up of 11 months over 3 years, prompting a national suspension of the procedure owing to concerns over its safety [11]. This rate was nearly double that reported in the COLOR II trial. Similarly, a comparative analysis of early cases revealed higher 2-year LR rates in robotic TME (9.5%) compared with LapTME (5.6%) [41]. These elevated rates were likely influenced by technical inexperience during the learning curve and suboptimal implementation. As newer techniques, TaTME and robotic TME inherently face challenges in early outcome evaluations owing to limited surgeon experience. As expertise improves, especially in challenging pelvic anatomy or advanced tumors, subsequent studies are better equipped to demonstrate their advantages.

The TaLaR trial reported comparable 3-year overall survival (OS), disease-free survival (DFS), and LR rates between TaTME and LapTME in patients with stage I–III mid-to-low rectal cancer [42]. Similarly, our analysis showed equivalent long-term oncologic outcomes among the three approaches. Importantly, 5-year oncological outcomes—including OS, DFS, LR, and DM rates—were comparable across all groups. These findings align with multiple RCTs and meta-analyses demonstrating consistent histopathological and survival outcomes across techniques [7, 12, 21, 42–44]. One meta-analysis using multivariate Cox regression found no significant differences in outcomes between the three approaches [43]. Similarly, a large multicenter PSM study using Cox regression confirmed that surgical technique did not predict OS or DFS [12].

Despite the significant findings, this study has several limitations. First, it was not a randomized trial and may be subject to selection bias and unmeasured confounding factors. Although PSM helped mitigate bias, residual confounders cannot be entirely excluded. In this report, although tumor location was adjusted, the average tumor distance from the anal verge was not adjusted using propensity score matching (PSM). Despite a statistically significant difference in tumor distance among the groups, the mean distances in all three groups still fall within the range of the mid-rectum (6 cm, 7 cm, and 8 cm in the TaTME, Robotic LAR, and LapTME group, respectively). Therefore, the influence of tumor distance from the anal verge is unlikely to outweigh the impact of tumor location on prognosis. Second, the study was conducted at a single center, potentially limiting the generalizability of the results to other healthcare settings and populations. Additionally, the sample sizes in the TaTME and robotic TME groups were relatively small, particularly after PSM, which reduced statistical power and limited the ability to detect meaningful differences in outcomes. Nevertheless, this study is among the few that compare laparoscopic, transanal, and robotic TME approaches. Research specifically addressing long-term oncological outcomes—especially for TaTME and robotic TME—remains limited. The short follow-up durations in previous studies highlight the need for continued surveillance and prospective randomized controlled trials. Our extended follow-up period offers a more comprehensive evaluation of long-term outcomes across minimally invasive techniques, providing valuable insights to support informed clinical decision-making.

## Conclusions

All three minimally invasive approaches—LapTME, TaTME, and robotic TME—are safe and feasible surgical options for rectal cancer. Although LapTME was associated with higher rates of overall postoperative complications and CRM positivity, these did not translate into inferior short- or long-term outcomes. The surgical approach should be tailored to the surgeon’s expertise, institutional resources, and patient-specific factors to achieve optimal outcomes.

## Data Availability

No datasets were generated or analyzed during the current study.
